# Practice of smoking cessation counselling and its associated factors among government primary healthcare workers in Perak: A cross-sectional study

**DOI:** 10.51866/oa.943

**Published:** 2026-01-24

**Authors:** Chang Hoong Lee, Anusha Vijayan, Dharishini Priya Nadarajan, Ibrahim Haniff Aizuddin, Ilavarase Nadraja, Karti Kunasekaran, Muhammad Khatib Haji Kamarul Ariffin, Khairatul Nainey Kamaruddin, Ai Theng Cheong

**Affiliations:** 1 Klinik Kesihatan Bagan Serai, Pejabat Kesihatan Daerah Kerian, Perak, Malaysia.; 2 Klinik Kesihatan Taiping, Pejabat Kesihatan Daerah Larut, Matang & Selama, Perak, Malaysia.; 3 Klinik Kesihatan Teluk Intan, Pejabat Kesihatan Daerah Hilir Perak, Perak, Malaysia.; 4 Klinik Kesihatan Simpang, Pejabat Kesihatan Daerah Larut, Matang & Selama, Perak, Malaysia.; 5 Klinik Kesihatan Batu Kurau, Pejabat Kesihatan Daerah Larut, Matang & Selama, Perak, Malaysia.; 6 Klinik Kesihatan Lekir, Pejabat Kesihatan Daerah Manjung, Perak, Malaysia.; 7 Klinik Kesihatan Manjoi, Pejabat Kesihatan Daerah Kinta, Perak, Malaysia.; 8 Department of Primary Care Medicine, Faculty of Medicine, Universiti Teknologi MARA, Sungai Buloh, Selangor, Malaysia.; 9 Department of Family Medicine, Faculty of Medicine and Health Sciences, Universiti Putra Malaysia, Serdang, Selangor, Malaysia.

**Keywords:** Smoking cessation, Counseling, Primary health care

## Abstract

**Introduction:**

Primary healthcare workers are in a strategic position to promote smoking cessation due to their accessibility. This study aimed to determine smoking cessation counselling (SCC) practice among primary healthcare workers and identify its associated factors.

**Methods:**

A cross-sectional online survey was conducted among 363 primary healthcare workers in all government healthcare clinics in the districts of Hilir Perak, Kerian, Kinta, Larut, Matang and Selama, and Manjung from January to June 2023. Doctors, allied health professionals, pharmacists, medical assistants, and nurses were included. Knowledge, attitude, and practice ofSCC were assessed using a validated 22-items questionnaire that covered 5 As (ask, advise, assess, assist and, arrange) and 5 Rs (relevance, risks, rewards, roadblocks and, repetitions). Multiple logistic regression was used to analyse the factors associated with poor practice.

**Results:**

Most participants were doctors (31.1% ), followed by allied health professionals (21.8%), pharmacists (19.3%), medical assistants (14.3%), and nurses (13.5%). The majority showed poor practice (93.7%), knowledge (94.8%), and attitude (51.5%). Only 16.8% were trained in SCC. Multivariate logistic regression analysis revealed that only the district was significantly associated with poor counselling practice. Hilir Perak district showed the highest odds of having poor practice (adjusted odds ratio (aOR) =17.80, 95% confidence interval (CI)= 2.02–156.97, P-value=0.01). However, the prevalence of poor practice among the other districts was also high (77.0%–97.8%). Conclusion: SCC practice, knowledge, and attitude are poor among Perak’s government primary healthcare workers. The district-specific differences suggest that localized studies should be considered to determine the influential factors.

## Introduction

Tobacco use is among the leading preventable causes of death and disease globally. It is estimated that more than 24,106 Malaysian deaths annually are related to smoking.^1^ In addition, a recent study conducted by Tan et al. found that smoking had serious negative impacts on health and work productivity among Malaysian adults.^2^ Smoking caused RM 275.3 billion loss of productivity, 5.5 million quality-adjusted life years and 3 million disability-adjusted life years.^2^ According to the National Health and Morbidity Survey (NHMS) 2023, the overall proportion of current tobacco smokers among Malaysians aged 15 years and above was about 19.0%.^3^ The proportion of current smokers was more than 39 times larger among men than among women.^3^ It only slightly decreased from 21.3%^4^ in 2019 to 19.0%^3^ in 2023.

Malaysia is a party to the World Health Organization Framework Convention on Tobacco Control (WHO FCTC), which requires its states parties to promote smoking cessation and provide treatment for smoking dependence.^5^ The Ministry of Health (MOH) Malaysia aimed to reduce smoking prevalence among adults aged 15 years and above in Malaysia to less than 15% by 2025, which is in alignment with the WHO FCTC.^6^ A holistic and structured programme known as the Malaysia Quit or mQuit service was developed under the National Strategic Plan on Tobacco Control to achieve the target. To date, 764 government healthcare clinics and hospitals have become mQuit providers.^7^ A clinical practice guideline on the treatment of tobacco use disorder was updated in 2016 to guide mQuit services across government and private practices. Effective counselling and pharmacological strategies are now the pillars of tobacco cessation programmes, especially when used in combination.^8^

SCC incorporates the 5 A and 5 R components.^6^ The 5 A components consist of ask, advice, assess, assist and arrange, which are useful when encountering any new patients, while the 5 R components comprise relevance, risks, rewards, roadblocks and repetitions, which are beneficial in convincing unmotivated smokers to stop smoking.^6^ SCC can be delivered not only by physicians but also by any medical personnel who have been trained formally.

Cochrane reviews have provided concrete evidence that stopping smoking could reduce smoking-related diseases.^9^ However, despite the abundance of smoking cessation programmes and quit smoking clinics (QSCs) established in Malaysia, the proportion of current smokers who attempted to quit smoking dipped slightly from 52.3% in 2015 to 48.9% in 2019.^3^

Primary healthcare workers (HCWs) are in a strategic position to help patients in smoking cessation.^10^ According to the guidelines for smoking cessation services at healthcare clinics by the MOH Malaysia, smoking cessation services in government primary healthcare clinics are run by multidisciplinary teams consisting of family medicine specialists (FMSs), medical officers, pharmacists, nurses and paramedics.^11^ Additionally, other clinical HCWs in healthcare clinics (e.g. clinical psychologists, dietitians, nutritionists, occupational therapists, counsellors and physiotherapists) can also provide SCC. Evidence suggests that SCC delivered by any healthcare provider improves the quit rate compared with no intervention.^12^ The literature also shows that SCC by multiple health professionals can substantially increase quitting and readiness to quit.^13^ A United Kingdom study showed that SCC provided by nurses and auxiliary healthcare providers was equally effective.^14^ Hence, to tackle smoking behaviours, all clinical HCWs should be actively involved in SCC.

According to the NHMS 2019, only three-quarters (77.3%) of current smokers who visited healthcare centres were advised to quit smoking by HCWs.^4^ Many factors were identified as barriers to effective SCC, some of which included a lack of knowledge; negative attitude; lack of training; lack of time, manpower and finance; and perception of insufficient patient motivation.^15^

Locally, a study in 2016 conducted among doctors in Kuala Lumpur found that 57.4% of government primary care doctors demonstrated poor SCC practice.^16^ In March 2021, another study conducted in Selangor investigated the knowledge, attitude and practice of SCC among primary HCWs.^17^ The authors used a self-developed questionnaire that assessed HCWs’ knowledge on the health effects of smoking and second-hand smoke exposure rather than knowledge on SCC.^17^ They did not focus on assessing SCC practice based on the main domains of the 5 As and 5 Rs of the smoking cessation framework.^17^ Conversely, a study that used a questionnaire covering the 5 As and 5 Rs in Selangor showed that 75.7% of government primary care doctors had poor SCC practice.^18^ This indicates a research gap in the assessment of SCC practice based on the 5 A and 5 R framework among primary HCWs who could engage with patients clinically for smoking cessation.

According to the NHMS 2019, the average proportion of current smokers in Malaysia who attempted to quit in the past 12 months was 48.9%, with the highest noted at Negeri Sembilan (62.2%) and the lowest at Pulau Pinang (24.5%). Perak ranked 11th among 16 states (43.4%) in the list. This shows that more effort is needed to improve smoking cessation services in Perak.^4^ Thus, this study aimed to assess the practice of SCC and its associated factors among primary HCWs in Perak. Involving all primary HCWs in our study allowed us to obtain a more comprehensive overview of the practice of SCC among HCWs in primary healthcare clinics in Perak.

## Methods

### Study design and setting

This cross-sectional study was conducted from January to June 2023 among government primary healthcare clinics in five districts of Perak. The five districts involved were Kerian; Larut, Matang and Selama; Kinta; Manjung; and Hilir Perak. These five districts were purposively selected, as they are the most populated districts in Perak, in which a large number of doctors serve. All categories of clinical HCWs who could engage with patients for smoking cessation were included in this study. They included doctors, medical assistants, nurses, pharmacists, pharmacy assistants, psychology officers, nutritionists, dietitians, occupational therapists and physiotherapists at the participating primary healthcare clinics. HCWs who had at least 6 months of working experience in primary care settings were included in the study, while those who were recruited for the pilot study, under a gazettement period and on a long leave during the study period were excluded.

### Sample size

The sample size was calculated based on the study by Rahmah et al.^16^ For identifying the associated factors, the sample size was determined using a 95% CI and a 5% margin of error, applying the formula for estimating proportions between two populations^19^ based on the proportions of doctors who exhibited poor SCC practice with (49.4%) and without (75.7%) previous SCC training. According to this calculation, the sample size for estimating the proportion of HCWs with poor SCC practice was 52 for each arm. The single-proportion formula was used to assess the practice of SCC among government primary HCWs based on the finite sample of 2609 HCWs from the five districts in Perak, and the estimated sample size was 329 based on a 57.4% proportion of primary care doctors with poor SCC practice and a 95% CI. A minimum total of 411 participants were required after accounting for a 25% non-response rate. Hence, we recruited 420 participants for the study, based on the highest sample size achieved.

### Sampling

Approval letters from the Medical Research Ethics Committee of the MOH and District Health Office (DHO) and WhatsApp invitation messages regarding this study were circulated through medical officers-in-charge (MOICs) at all healthcare clinics in the five districts to inform all HCWs of the study. HCWs were advised that they may contact the human resource department if they do not wish to disclose their contact details. Thereafter, lists of the phone numbers of all government primary HCWs were acquired through the human resource department from each DHO. Participants were selected via stratified random sampling. They were stratified into the following four groups: doctors, nurses/medical assistants, pharmacists/assistant pharmacists and others. Dietitians, nutritionists, psychology officers, physiotherapists and occupational therapists were classified into the group of ‘others’, as their number was small (60). Subsequently, an equal number of 120 participants from the other three groups were randomly selected using a research randomiser tool (Research Randomizer, 2022).^20^

MOICs from each healthcare clinic were approached and briefed. Participants who fulfilled the eligibility criteria were given messages via WhatsApp by MOICs with the invitation link on Google Forms together with participants’ unique code numbers. Selected participants were given an explanation regarding the study on Google Forms and provided their consent before proceeding to answer the questionnaire. No name, identification card number or phone number was required in the survey form to ensure confidentiality and mitigate response bias. Participants were not required to sign in to an account to fill in the survey. Participants’ code numbers were used as identifiers to prevent double-entry. WhatsApp reminder messages were sent to participants at the end of the first week when participants did not respond to ensure a high response rate. If there was still no response, follow-up reminders were sent in the middle of the second week. Those who were not responsive to the reminder messages within 2 weeks or did not consent to participate in the study were considered non-responders.

### Research tools

The online questionnaire was created on Google Forms. There were two sections in the questionnaire, whereby the first section examined the sociodemographic characteristics of participants (age, sex, ethnicity, district name, profession and smoking status). This section also evaluated the number of smokers seen per week, clinic led by an FMS, SCC training status and QSC involvement.

The second section assessed the knowledge, attitude and practice of HCWs using a locally validated questionnaire.^21^ This questionnaire was validated among primary care doctors. It could also be used to assess other support medical staff trained in smoking cessation training programmes. It consisted of three components: knowledge, attitude and practice of SCC. There were a total of 22 items, with five items on knowledge, five items on attitude and 12 items on practice.

The knowledge component was assessed utilising a dichotomous scale consisting of options of ‘true’ and ‘false’. A score of 1 was given for a correct answer and 0 for an incorrect answer. The attitude component was evaluated based on the degree of agreement, using a 5-point Likert scale: ‘strongly agree’, ‘agree’, ‘do not know’, ‘disagree’ and ‘strongly disagree’. The items were scored as follows: 1 for ‘disagree’, 2 for ‘strongly disagree’ and 0 for the other responses (‘strongly agree’, ‘agree’ and ‘do not know’). For items 8 and 10, reverse coding was applied: 1 for ‘agree’, 2 for ‘strongly agree’ and 0 for the other responses (‘strongly disagree’, ‘disagree’ and ‘do not know’). The practice component was measured using a 4-point Likert scale as follows: 2 for ‘always’, 1 for ‘frequent’ and 0 for the other responses (‘seldom’ and ‘never’).

The practice score ranged from a minimum score of 0 to a maximum score of 24. Practices were categorised into two groups: the good practice group for participants who scored the full 24 marks and the poor practice group for those who scored less than 24 marks. The total possible knowledge score was 5. Knowledge was categorised as good (score of 5) or poor (score of <5). The attitude score ranged from 0 to 10. Participants who scored >5 marks were categorised to exhibit good attitudes, while those who scored <5 marks were deemed to demonstrate poor attitudes.^21^

Permission was taken from the author to use the questionnaire. For this study, the questionnaire was translated from English to Malay and then from Malay back to English by two groups of medical assistant clinical instructors from Kolej Sains Kesihatan Bersekutu Sultan Azlan Shah. A pilot study was conducted among 42 primary HCWs from the five districts in Perak to validate the translated questionnaire. Participants were selected via convenience sampling. There were 13 doctors, 10 medical assistants, eight staff nurses, nine community nurses and two pharmacists involved. They were excluded from the actual study sample. A high degree of internal consistency was observed, with an overall Cronbach’s alpha value of 0.92 and good knowledge (0.72), attitude (0.81) and practice subscales (0.94).

### Data analysis

Data were analysed using IBM SPSS Statistics version 29.0 (SPSS Inc., Chicago, IL). The dependent variable in this study was the level of practice of SCC. The independent variables were knowledge of SCC, attitude towards SCC, age, sex, smoking status, number of smokers seen per week, district, clinic led by an FMS, profession, SCC training status and QSC involvement.

The Pearson chi-square test or Fisher’s exact test was used for bivariate analysis, and multiple logistic regression was used for multivariate analysis to examine the factors associated with the practice of SCC. Factors with P-values of <0.25 in the simple logistic regression were included in the multiple logistic regression. The results will be presented in tables. P-values of <0.05 were deemed to be statistically significant.

## Results

The response rate was 86.4% (363/420). Most participants were aged 30–39 years (61.7%) and were women (71.6%). Approximately 94.5% were non-smokers, and 73.6% reported seeing at least one smoker patient per week at work. Of the five districts, Kinta had the most participants (34.4%), while Manjung had the least (12.4%). Only a small proportion of the participants received SCC training through workshops/seminars (14.6%) or online programmes (2.2%) (Table 1).

**Table 1:** Sociodemographic characteristics of the participants (N=363).

Variable	Frequency (%)
**Age, year**	
<30	33 (9.1)
30-39	224 (61.7)
≥40	106 (29.2)
**Sex**	
Male	103 (28.4)
Female	260 (71.6)
**Ethnicity**	
Malay	261 (71.9)
Chinese	39 (10.7)
Indian	53 (14.6)
Others	10 (2.8)
**Smoking status**	
Non-smoker	343 (94.5)
Active smoker	20 (5.5)
**Number of smokers seen per week**	
0	96 (26.4)
≥1	267 (73.6)
**District**	
Hilir Perak	61 (16.8)
Kerian	61 (16.8)
Kinta	125 (34.4)
Larut, Matang and Selama	71 (19.6)
Manjung	45 (12.4)
**Clinic led by an Family Medicine Specialist (FMS)**	
No	71 (19.6)
Yes	292 (80.4)
**Profession**	
Doctor	113 (31.1)
Pharmacist	70 (19.3)
Medical assistant	52 (14.3)
Nurse	49 (13.5)
Allied health professional	79 (21.8)
**Smoking cessation counselling (SCC) training status**	
Attended a workshop or seminar	53 (14.6)
Attended an online programme	8 (2.2)
Not trained	302 (83.2)
**Quit smoking clinic (QSC) involvement**	
No	277 (76.3)
Yes	86 (23.7)

In the knowledge subscale, 73.0% and 63.9% of the participants attained incorrect answers for the assess and assign components. In the attitude subscale, only 55.1% of the participants disagreed with the item ‘I feel that my effort in helping smokers to quit is not well rewarded’. Conversely, the majority of the participants either agreed or strongly agreed that repetition in advising smokers is beneficial (80.2%) and that every HCW should be provided with an algorithm on treating chronic smokers (88.2%). In the practice subscale, Table 2 shows that less than half of the participants always advised smokers to quit (46.8%) and to reduce the number of cigarettes smoked per day (47.4%). Moreover, only 22.9% always practised the assist component of the 5 A approach, and 28.4% always conducted further follow-ups for smokers. Table 3 shows that the majority of the participants exhibited poor SCC practice (93.7%) and knowledge (94.8%), whereas more than half (51.5%) showed poor attitudes towards SCC.

**Table 2:** Responses to the questionnaire on SCC knowledge, attitude and practice (N=363).

Item no.	Question		Correct answer n (%)	Incorrect answer n (%)
Knowledge				
1	**Assess is the first component under the 5 As of the smoking cessation clinical practice guidelines.**		98 (27.0)	265 (73.0)
2	**Assign is one of the components under the 5 As of the smoking cessation clinical practice guidelines.**		131 (36.1)	232 (63.9)
3	**Assist is the subsequent component after advising patients to quit smoking.**		226 (62.3)	137 (37.7)
4	**The 5 Rs are used for those who are unwilling to quit smoking at any time.**		183 (50.4)	180 (49.6)
5	**Reusage is the last component of the 5 R framework in the smoking cessation clinical practice guidelines.**		205 (56.5)	158 (43.5)

Attitude				
Item no.	Question	Response, n (%)		
		0 (strongly agree/agree/do not know)	1 (disagree)	2 (strongly disagree)
6	I feel that my effort in helping smokers to quit is not well rewarded.	163 (44.9)	148 (40.8)	52 (14.3)
7	The clinical practice guidelines are not relevant in improving patient smoking cessation.	116 (31.9)	172 (47.4)	75 (20.7)
8	Repetition in giving advice on smoking cessation to patients is beneficial.	72 (19.8)	180 (49.6)	111 (30.6)
9	The framework for approaching chronic smoking is impractical.	161 (44.3)	156 (43.0)	46 (12.7)
10	Every provider should be provided with an algorithm on treating chronic smokers.	43 (11.8)	218 (60.1)	102 (28.1)

**Practice**				
Item no.	Question	Response, n (%)		
		0 (never/seldom)	1 (frequent)	2 (always)
11	I check when my patient last smoked.	120 (33.1)	99 (27.3)	144 (39.7)
12	I advise smokers to quit.	80 (22.0)	113 (31.1)	170 (46.8)
13	I advise smokers to reduce the number of cigarettes smoked per day.	71 (19.6)	120 (33.1)	172 (47.4)
14	I inquire about smokers’ willingness to quit.	97 (26.7)	113 (31.1)	153 (42.1)
15	I provide smokers with practical counselling.	180 (49.6)	100 (27.5)	83 (22.9)
16	I conduct further follow-ups with smokers who are quitting.	175 (48.2)	85 (23.4)	103 (28.4)
17	I encourage smokers to indicate why quitting is personally important.	127 (35.0)	118 (32.5)	118 (32.5)
18	I ask smokers to identify any potential harm of smoking to self.	117 (32.2)	120 (33.1)	126 (34.7)
19	I ask smokers to identify the negative consequences of continuing smoking.	126 (34.7)	104 (28.7)	133 (36.6)
20	I ask smokers to identify the advantages of quitting smoking to their family.	112 (30.9)	115 (31.7)	136 (37.5)
21	I ask smokers why quitting is impossible.	155 (42.7)	127 (35.0)	81 (22.3)
22	I continuously inform smokers about the benefits of quitting smoking.	92 (25.3)	124 (34.2)	147 (40.5)

**Table 3:** Levels of practice, knowledge and attitude towards smoking cessation counselling (N=363).

Variable	Frequency (%)
**Practice level**	
Poor	340 (93.7)
Good	23 (6.3)
**Knowledge level**	
Poor	344 (94.8)
Good	19 (5.2)
**Attitude level**	
Poor	187 (51.5)
Good	176 (48.5)

Table 4 shows that smoking status (P=0.030), district (P<0.001), QSC involvement (P=0.001) and profession (P=0.013) were significantly associated with the level of practice of SCC. Knowledge and attitude were not significantly related to practice.

**Table 4:** Bivariate analysis of the association of the SCC practice level with the sociodemographic factors, SCC training status, QSC involvement, knowledge level and attitude level (N=363).

Sociodemographic factor	Practice level, n (%)		Statistical test	P-value
	Poor(n=340)	Good (n=23)		
**Age, year**			1.77 (2)^a^	0.413
<30	31 (93.9)	2 (6.1)		
30-39	207 (92.4)	17 (7.6)		
>40	102 (96.2)	4 (3.8)		
**Sex**			2.76 (1)^a^	0.097
Male	93 (90.3)	10 (9.7)		
Female	247 (95.0)	13 (5.0)		
**Smoking status**				0.030^b^
Non-smoker	324 (94.5)	19 (5.5)		
Active smoker	16 (80.0)	4(20.0)		
**Number of smokers seen per week**			1.04 (1)^a^	0.309
0	92 ( 95.8)	4 (4.2)		
≥1	248 (92.9)	19 (7.1)		
**District**				<0.001^b^
Kerian	47 (77.0)	14 (23.0)		
Hilir Perak	60 (98.4)	1 (1.6)		
Kinta	121 (96.8)	4 (3.2)		
Larut, Matang and Selama	68 (95.8)	3 (4.2)		
Manjung	44 (97.8)	1 (2.2)		
**Clinic led by an FMS**				0.179^b^
No	64 (90.1)	7 (9.9)		
Yes	276 (94.5)	16 (5.5)		
**Profession**				0.179^b^
Doctor	101 (89.4)	12 (10.6)	8.61 (2)^a^	0.013
MA/nurse	93 (92.1)	8 (7.9)		
PF and allied health professional	146 (98.0)	3 (2.0)		
**SCC training**				0.246^b^
No	285 (94.4)	17 (5.6)		
Yes	55 (90.2)	6 (9.8)		
**QSC involvement**			14.64 (1)^a^	<0.001
No	267 (96.4)	10 (3.6)		
Yes	73 (84.9)	13 (15.1)		
**Knowledge level**				0.342^b^
Poor	323 (93.9)	21 (6.1)		
Good	17 (89.5)	2 (10.5)		
**Attitude level**			2.75 (1)^a^	0.097
Poor	179 (95.7)	8 (4.3)		
Good	161 (91.5)	15 (8.5)		

a Pearson chi-square test

b Fisher’s exact test, statistically significant

Table 5 demonstrates that only the district was significantly associated with poor SCC practice. Kerian was chosen to be the reference category, as it had the lowest prevalence of poor SCC practice among the districts. The other districts had 5.1–17.8 times higher odds of poor SCC practice than Kerian.

**Table 5:** Logistic regression analysis of the factors associated with a poor SCC practice level

Variable	Simple logistic regression		Multiple logistic regression	
	Crude OR (95% CI)	P-value	Adjusted OR (95% CI)	P-value
Age, year				
<30 30-39 >40	1 0.79 (0.17, 3.57) 1.65 (0.29, 9.41)	0.755 0.576	-	-
Sex				
Male	1		1	
Female	2.04 (0.87, 4.82)	0.103	1.31 (0.48, 3.60)	0.594
Smoking status				
Active smoker	1		1	
Non-smoker	4.26 (1.30, 14.00)	0.017	1.10 (0.23, 5.34)	0.907
**Number of smokers seen per week**				
≥1 0	1 1.76 (0.58, 5.32)	0.315	-	-
**District**				
Kerian	1		1	
Hilir Perak	17.87 (2.27, 140.84)	0.006	17.80 (2.02, 156.97)	0.010
Kinta	9.01 (2.82, 28.78)	<0.001	8.17 (2.13, 31.31)	0.002
LMS	6.75 (1.84, 24.80)	0.004	5.10 (1.22, 21.36)	0.026
Manjung	13.11 (1.65, 103.87)	0.015	9.43 (1.05, 84.94)	0.045
**Clinic led by an FMS**				
Yes	1.89 (0.75, 4.78)	0.180	1.88 (0.64, 5.52)	0.248
No	1		1	
QSC involvement				
Yes	1		1	
No	4.76 (2.00, 11.28)	<0.001	1.99 (0.61, 6.45)	0.254
**Profession**				
Doctor	1		1	
MA/nurse	1.38 (0.54, 3.53)	0.500	1.35 (0.46, 4.00)	0.585
PF and allied health professional	5.78 (1.59, 21.01)	0.048	3.22 (0.73, 14.18)	0.121
**SCC training**				
Yes	1		1	
No	2.37 (0.93, 6.04)	0.071	2.11 (0.62, 7.20)	0.234
**Knowledge level**				
Poor	1		1	
Good	0.55 (0.12, 2.55)	0.447	1.18 (0.20, 6.98)	0.856
**Attitude level**				
Poor	1		1	
Good	0.48 (0.20, 1.16)	0.103	0.46 (0.17, 1.27)	0.134

The bolded numbers are values that differed significantly at P<0.05 in the multiple logistic regression analysis.

## Discussion

This cross-sectional study examined the level of practice of SCC and its associated factors among government primary HCWs in five districts of Perak. Our results showed that the proportion of primary HCWs with poor practice and knowledge of SCC was substantially large at 93.7% and 94.8%, respectively, while nearly half (51.5%) showed poor attitudes towards SCC. The district was the only variable found to be significantly associated with SCC practice. The other sociodemographic factors, SCC training status, QSC involvement and SCC knowledge and attitude were not significantly related to SCC practice.

Less than 50% (39.7%–42.1%) of the participants consistently asked, advised on and assessed the smoking status of their patients; an even smaller proportion (19.7%–29.5%) assisted patients and arranged for appointments. These results are consistent with two reports from Europe showing an unsatisfactory practice of healthcare professionals, with 36.6%–51.9% asking, advising on and assessing patients; 19.7%–29.5% assisting patients; and 9.4%–14.9% arranging for appointments.^22,23^ These results imply that most HCWs tend to provide assistance and arrange for follow-up among patients interested in quitting. In addition, we found that the practice of the 5 Rs was poor, as only 22.3%–40.5% of the HCWs had always practised them. These findings are similar to another report in Selangor among primary care doctors (14.1%–26.1%).^18^

The proportion of doctors with poor SCC practice (89.4%) is much larger in our study than in other studies conducted among primary care doctors in Kuala Lumpur (57.4%),^16^ Selangor (50.9%),18 Saudi Arabia (52.7%)^24^ and Egypt (56%).^25^ These findings could be explained by the strict scoring criteria of the questionnaire used in this study that required the participants to score full marks in all 12 practice component items to be considered as having good SCC practice. We could not find other studies that used the same scoring criteria for categorising good and poor practices. Other Malaysian studies utilised score ranges for categorisation of good and poor practice levels.^16,18^ In some other studies, participants with scores equal or above the mean scores were considered as having good practice levels.^24,25^

The proportion of HCWs with SCC training was small (16.8%), consistent with the findings of another study in Selangor (14.1%)^17^ that was also conducted among various types of primary HCWs. The large proportion of HCWs untrained in SCC (83.2%) could limit the validity of the questionnaire used, as it was found suitable to assess those who had been trained in smoking cessation training programmes.^21^ Additionally, the inclusion of HCWs from maternal and child health clinics might explain the much poorer SCC practice and knowledge in our study, as they may not be aware of their role in SCC due to the small proportion of smokers among women^26^ and children. This low awareness needs to be addressed, as smoking is prevalent among 1.4% of antenatal mothers, of whom 42.4% are still smoking at their first antenatal visit.^27^ Smoking status should be assessed among pregnant mothers and older children as well as the accompanying spouse or parent.

Despite the existence of QSCs in all government primary clinics, enrolment remains low. One way to increase enrolment rates is to promote the practice of the 5 As among all frontline staff. Although the levels of knowledge and attitude were low among most HCWs, they were not found to be a significant factor related to SCC practice, in contrast to few reports in Malaysia.^16-18^ A Kuantan study also demonstrated that FMSs, HCWs with previous SCC training and HCWs in charge of QSCs showed better SCC practice.^28^ These aspects require further exploration to identify the barriers to SCC practices among HCWs.

In this study, one district stood out from having poor SCC practice. This implementation gap could be due to reasons at different levels such as discrepancies in organisational support, patient-to-HCW ratio, availability of health promotion materials and nicotine replacement medication.^22^ Therefore, further study is needed to assess the status and association of the abovementioned factors with SCC practice in Perak. However, there is room for improvement for all five districts, as the prevalence of poor SCC practice remains high, ranging from 77.0% to 98.4%.

This study provides insights for practice implications. There was a significant difference found in SCC practices between the districts. Therefore, SCC training should be given to all frontline staff in all districts. This study also suggests that interventions to improve SCC practice should not focus merely on training for knowledge and attitude.

This study covered most categories of clinical-based primary HCWs in government healthcare clinics in Perak. However, it may not be representative enough to attribute the findings to nationwide government and private primary HCWs. This is because the resources and situations in each state and private settings might be different. In addition, the use of a self-administered questionnaire may be subject to recall bias, leading to under- or over-reporting. The attitude and practice components were recategorised from 5-point and 4-point Likert scales into 3-mark categories and then into two-outcome categories (good and poor). This process made the analysis easier to understand. However, it may have lost the differences between participants in the initial Likert scale measurements, resulting in under- or overestimation of those in the poor and good categories. Several factors of SCC practice were not included in the questionnaire such as language barrier, organisational support, patient-to-HCW ratio, availability of health promotion materials and specialised QSCs. Future research could be strengthened by incorporating these factors into the questionnaire.

## Conclusion

SCC practice, knowledge and attitude are poor among primary HCWs in Perak government healthcare clinics. The low rate of formal training underscores the need for targeted interventions. The district-specific differences suggest that localised studies should be considered to determine the influential factors.

## Data sharing statement

The data collected for this study are available from the corresponding author upon request.

**How does this paper make a difference in general practice?**

- This study highlights the substantially high prevalence of poor smoking cessation counselling (SCC) practice among primary healthcare workers in government clinics.
- SCC can be conducted by all frontline healthcare workers to optimise patients’ exposure towards smoking cessation services.
